# Exploring the experience of appetite loss in older age: insights from a qualitative study

**DOI:** 10.1186/s12877-024-04732-9

**Published:** 2024-01-31

**Authors:** Lorelle Dismore, Avan Sayer, Sian Robinson

**Affiliations:** 1 AGE Research Group, Translational and Clinical Research Institute, Faculty of Medical Sciences, Newcastle University, Newcastle Upon Tyne, UK; 2 Northumbria Healthcare NHS Foundation Trust, Research and Development, North Tyneside General Hospital, North Shields, UK; 3 NIHR Newcastle Biomedical Research Centre, Newcastle University, Newcastle upon Tyne NHS Foundation Trust and Cumbria, Northumberland, Tyne and Wear NHS Foundation Trust, Newcastle Upon Tyne, UK

**Keywords:** Appetite, Ageing, Under-nutrition

## Abstract

**Background:**

Poor appetite is common in older age, with estimated prevalence figures ranging between 15–30% in community-dwelling populations. Despite known links between poor appetite and adverse health outcomes, appetite is not routinely assessed and the causes and impact of appetite loss in older age are not well understood. This study aimed to improve understanding of the influences on, and experiences of, appetite loss among older people who have poor appetite and to consider the implications for prevention and treatment strategies.

**Methods:**

Thirteen older adults aged 60–93 years (9 women) identified as having poor appetite (Simplified Nutritional Appetite Questionnaire (SNAQ) scores < 14; ranging from 8–11) took part in semi-structured interviews. Open-ended questions focused on influences on and experiences of appetite and appetite loss in older age. Interviews were transcribed; reflective thematic analysis was conducted using an inductive approach.

**Results:**

The analysis generated three themes: 1) a complex web of influences on appetite loss, that include biological, psychological, and social factors; 2) living with poor appetite—variation in perceptions of poor appetite and attitudes to appetite loss; 3) living with poor appetite—the role and importance of the eating environment and social interactions. The themes highlight marked differences in individual ‘journeys’ to poor appetite, with variation in the balance and role of specific causal influences, that impact on the experience of appetite loss and on coping strategies.

**Conclusions:**

A broad range of influences (including biological, psychological and social factors) and experiences of appetite loss in older age were described. Future research should consider person-centred approaches, that take account of individual narratives of appetite loss, in the design of effective strategies to support older adults.

**Supplementary Information:**

The online version contains supplementary material available at 10.1186/s12877-024-04732-9.

## Background

Appetite is not routinely assessed in clinical practice despite poor appetite being commonly reported in older age, with estimated prevalence figures between 15–30% in community-dwelling populations [1]. Understanding of appetite loss remains limited although, to an extent, seen by many older adults as an expected feature of ageing, arising from the effects of changes in vision, taste and sense of smell [2–4], alongside age-related changes in gut function, biting/chewing problems and the effects of illness and medication [3–7]. Additionally, a range of other influences on appetite loss have also been reported that include poor psychological health and social changes in later life, such as living and eating alone [3, 7, 8].

Poor appetite can lead to inadequate dietary intake, both in the amount of food consumed as well as having negative effects on dietary diversity and diet quality [7, 9]. The consequent increased risks of under-nutrition, weight loss and muscle loss are associated with worse health outcomes that include sarcopenia, frailty and greater mortality [7, 10–12]. Detecting loss of appetite before weight loss and dietary changes occur is needed if early intervention and potential prevention of a decline in health are to occur [4]. However, despite growing evidence of the role of specific risk factors, the experience of appetite loss in older age remains poorly described [13]. To date, there has been limited qualitative research in this field and, so far, mainly focused on hospitalised and malnourished patients, for whom appetite changes may be impacted by health conditions and acute illness [14, 15]—potentially providing limited insights into the experience of appetite loss for the majority of older adults who are outside these settings.

A better understanding of appetite loss is needed to inform the design of preventive strategies, as well as treatment options, for which there are few at present [13, 16]. To address current gaps in knowledge, this study used qualitative methodology to enable an exploration of the perspectives of older adults who live with poor appetite, their experiences of appetite and appetite loss, and the contexts of those experiences. The aim of our study was to improve understanding of appetite loss in older age and to consider the implications for prevention and treatment opportunities.

## Methods

### Study design

Our study was carried out in two stages. In the first stage of the study, we worked with a group of older adults (the Patient and Public Involvement (PPI) group) to discuss issues around appetite in older age. These discussions informed the design of the research and the plans for individual interviews with older adults who had poor appetite about their experiences (study participants) in the second stage of the study. Thematic analysis was applied to the semi-structured interviews. Ethical approval was granted from Newcastle University Ethics Committee (No. 923).

### Participants and recruitment

Members of the PPI group were recruited via VOICE (https://www.voice-global.org/)—a large citizen network, with reach across the United Kingdom (UK) and internationally, that enables a wide range of individuals to contribute their experiences and perspectives to develop research & innovation in health and ageing. An opportunity to participate in the PPI group was advertised online via the VOICE website. Ten older adults (six women, four men; age range 57–87 years) volunteered to take part in a series of PPI group meetings over the period of the study. In the first meeting (June 2021) we discussed issues of appetite loss and how to reach older adults with poor appetite to invite them to participate in the research study. The second PPI group meeting (July 2021) included a focus group discussion of the factors influencing appetite decline. The findings from the focus group informed the development of the interview schedule for the individual participant interviews in the second stage of the study. The discussions were led by an experienced health psychologist who has expertise in qualitative methods (LD).

In the second stage, study participants were recruited in a separate phase of recruitment via VOICE. An ‘opportunity’ to participate in the research was published in July 2021. Potential participants were invited to complete an on-line version of the Simplified Nutritional Appetite Questionnaire (SNAQ) [17], taking approximately 5–10 minutes to complete, and to provide demographic details, including their name, age and gender. Individuals were eligible to take part in the interview in the second stage of the study if they were aged over 60 years and had a SNAQ score of < 14, indicating they had poor appetite.

After removing duplicate questionnaires, there were 59 potential participants who had completed the SNAQ screening. Forty-four of these were not eligible to take part in the second stage of the study (they had a SNAQ score of $\ge$ 14 or were aged < 60 years); of the remaining 15 potential participants who were identified as eligible, 13 took part in an interview in the second stage of the study. The 13 participants who took part in semi-structured interviews were aged between 60–93 years and included nine women and four men. Ten participants were White British, three were British Asian: seven lived alone, six with a spouse. SNAQ scores ranged between 8–13. The number of daily medications ranged from 1–21; six participants had lost weight unintentionally in the previous 12 months.

### Interviews

Data were collected over the period August-December 2021 via in-depth semi-structured interviews led by one researcher (LD); the participants had not met the researcher before. Each participant provided witnessed consent prior to their interview over a telephone/video call. The researcher read out the statements on the consent form to each participant to obtain their verbal agreement, with a witness present. Once confirmed that the participant agreed with the statements, the consent form was signed by the researcher and by the witness.

Eight participants were interviewed via an on-line video call, and five participants were interviewed over a telephone call; all participants were in their homes during the interviews. Each interview lasted on average 60 minutes. For one interview with a participant who had a hearing problem, a family member was present during the interview. The family member clarified or re-iterated some of the questions for the participant, but only participants’ responses were used in the analysis.

The interview schedule utilised open-ended questions and was designed based on published literature and informed by the focus group discussion with the PPI group. For example, this discussion highlighted the need to explore the impact of the social context of eating and emotional well-being. Open-ended questions focused on participants’ experiences of appetite loss and factors they perceived that influenced their appetite, ‘Can you tell me about your appetite?’ and ‘What are the reasons you feel you may have poor appetite?’. The interview schedule is given in Supplementary Table 1. The interviews were audio-recorded and then transcribed verbatim. All interview transcripts were anonymised before analysis. Interviews were continued until sufficient data had been collected, judged as providing meaningful answers to the study questions.

### Data analysis

Interview transcripts were analysed by the first author (LD) using Braun and Clark’s [18, 19] reflexive thematic analysis that provides a rich, detailed and complex account of data using an accessible and theoretically flexible approach. This process identified initial codes from the transcripts using a coding framework that was developed to represent emerging themes. The thematic analysis was applied using an inductive approach, with codes grounded in the content of the data and driven by participant’s language and concepts, rather than using a pre-existing coding frame [20]. The steps involved reading and re-reading each data transcript to ensure familiarisation. Whilst reading over the transcripts, the initial codes generated through identifying interesting aspects of the data were captured in written notes. Once codes were established, they were collated into potential themes. The authors met regularly to discuss the themes that were constructed by the researcher’s (LD) subjectivity and an interpretative reflexive process. To aid the thematic analysis, selected participant quotations were presented to the PPI group at their third meeting for discussion and interpretation. The PPI group were asked about their impressions and reflections on the quotations and their feedback was sought on the potential themes. Members of the PPI group had diverse experiences of appetite loss; their input and perspectives guided the development and confirmation of the potential themes. These were then further discussed with all authors and refined. The themes were identified to form a coherent and analytic narrative of the phenomena, and the quotations that best captured the themes were selected for illustration.

## Results

The thematic analysis generated three themes, the first centred on appetite loss: 1) a complex web of influences on appetite loss, that include biological, psychological, and social factors; with the other two centred on the lived experience: 2) living with poor appetite—variation in perceptions of poor appetite and attitudes to appetite loss; 3) living with poor appetite—the role and importance of the eating environment and social interactions. The themes are described below and illustrated with representative participant quotes, together with details of their gender, age and SNAQ score. A summary of the themes is provided in Table 1.

**Table 1:** Summary of themes with representative quotations

**1) A complex web of influences on appetite loss, that include biological, psychological and social factors**	
***- Biological***	*“I don’t get up tomorrow morning and go and dig holes in the roads, I don’t need the energy, I just need to fill the gap occasionally”* (Male, 79 years, SNAQ: 13)
***- Psychological***	*“I’ve always had mental health issues…my mental health took a turn for the worst in 2008 …and since then my appetite has got worse, my mental health has got worse which has impinged on my food intake and my appetite”* (Male, 61 years, SNAQ: 12)
***- Social***	*“We were in lockdown…it was a complete change of lifestyle”* (Female, 76 years, SNAQ: 10)
**2) Living with poor appetite—variation in perceptions of poor appetite and attitudes to appetite loss**	
***- Poor recognition or acceptance***	*“I would say prove it, tell me why, tell me how, compared with what?”* (Male, 79 years, SNAQ: 13)
***- Eating to survive***	*“I never feel like I’m not eating enough because I’m surviving”* (Female, 66 years, SNAQ: 12)
***- Practical strategies***	*“I like to have small portions during the course of the day so…or like a snack rather than have one big meal in the evening”* (Female, 66 years, SNAQ: 13)
**3) Living with poor appetite—the role and importance of the eating environment and social interactions**	
***- Eating environment***	*“I think that if I eat with somebody else, I more enjoy myself than on my own… that’s why I’ve got one or two friends and then when I’m out with them I have a proper meal, I eat with my friend or I eat in a restaurant and I feel like, you know I’m happy”* (Female, 60 years, SNAQ: 11)
***- Social context***	*“If I’m going out for a meal with friends then I eat what I like, I can eat three courses no problem”* (Female, 76 years, SNAQ: 10)

### Theme 1: a complex web of influences on appetite loss, that include biological, psychological, and social factors

Individual ‘journeys’ to poor appetite were complex, influenced by a range of biological, psychological, and social factors that differed between participants in their balance and impact. Appetite loss was commonly attributed to a combination of influences from the three domains and were often interrelated.

#### Biological factors

The participants described age-related changes in appetite and the effects of changes in their lifestyle and routines in older age, specifically linking lower levels of physical activity to a decreased need for energy.

*“It has reduced over the years; I think I definitely eat less now than even five years ago because…part of that is that I don’t do as much”* (Male, 93 years, SNAQ: 11)

Daily tasks were emphasised as taking longer to perform and causing fatigue. Eating food was no longer prioritised because of the importance of managing this around routines.

*“I’ve had a busy morning… I haven’t had anything to eat, I haven’t had a lunch today…and I won’t have anything to eat until teatime…”* (Male, 79 years, SNAQ: 13)

Age-related changes in appetite were also linked to experience of acute illness and long-term health conditions and to the use of medication.

*“Certainly, the Loratadine is designed to affect my appetite…they use it for people with obesity to try and regulate their appetite, but mine was used as a diabetic drug, quite a few years ago …I think the diabetic drugs are the main ones that have an effect on my appetite”* (Female, 66 years, SNAQ: 12)

#### Psychological factors

Emotional well-being was often linked to appetite, with some participants attributing the loss of motivation and desire to eat to their mental health issues.

*“I put it down to my mental health issues as well sort of, not be bothered of actually doing things in general… it’s just sometimes it’s too much effort to actually get up and do something like that…it annoys me, even when I’m going through a bad patch, I should eat something really, but I can’t be bothered…when I’m going through a good patch my appetite…is good…I eat more and eat better”* (Male, 61 years, SNAQ: 12)

Health problems and psychological issues frequently co-existed, such as living with long term conditions and adjusting to coping with health problems that prompted negative psychological reactions leading to appetite loss and a reduced desire to eat. For example, participants described feeling embarrassed when eating with others due to a tremor in the hand or experiencing difficulties with swallowing, making it preferable to eat alone or only with close relatives. For some participants, eating was no longer a pleasurable experience.

*“I think it has changed over time… the change I think over a few years now… I think it’s a gradual change since I’ve been diagnosed with diabetes and also…depression and my mental health”* (Female, 60 years, SNAQ: 11)

#### Social factors

Social interaction around meals and food preparation was identified by many participants as an important positive influence on appetite—with appetite loss described in relation to changes in social factors, such as the loss of routine following retirement from work, or the effects of living and eating alone.

*“I think also cooking for oneself goes, that adds onto the range of what one’s eating…is the lack of cooking…I vary rarely cook or bake as I used to…I was always…with family but on my own, no, it becomes worse as one gets older…”* (Female, 82 years, SNAQ: 12)

Participants highlighted the negative impact of the UK ‘lockdown’ during the COVID-19 pandemic which prevented opportunities for social interaction outside the home in the period 2020–2021. Although the official lockdown had ended several months before the participants were interviewed in this study, there was still some ongoing impact, with continuing caution around social interactions advised for higher-risk groups, that included older adults.

**Fig. 1:**
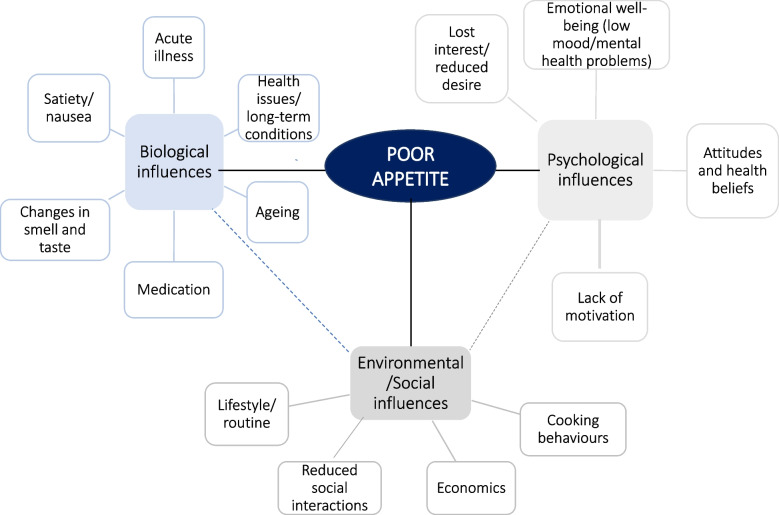
A complex web of influences on appetite loss. Influences are categorised as biological, psychological, and social but are interrelated – indicated by the dotted lines

The complexity of the web of potential influences on appetite loss described by the participants is represented in Fig. 1, underlining the differences in individual ‘journeys’ to poor appetite. Although these influences could be grouped into biological, psychological, and social factors, in reality they were overlapping and interrelated; for example, with one participant describing how her physical state impacted on her social activities which, in turn, caused her poor psychological well-being, and contributing to her appetite loss.

### Theme 2: living with poor appetite—variation in perceptions of poor appetite and attitudes to appetite loss

Although all participants in the study had poor appetite, there were notable differences in their perceptions of appetite loss and their attitudes to it.

#### Poor recognition or acceptance

Some participants did not recognise that they had poor appetite.

*“I mean I’ve been told by other older ladies, as you get older you do lose your appetite a little bit, but I just don’t know whether I have or not…will it impact me a bit further or not? I don’t know…I don’t know why I’m changing…I have no idea…food still smells delicious, but I don’t want to eat it”* (Female, 66 years, SNAQ: 9)

Others were reluctant to acknowledge their appetite loss.

*“I’m satisfied I eat enough… I don’t think it would cause me to be worried if I had to do without food for a couple or three days, I don’t just think I would disappear…. How do you define without and reduced?”* (Male, 93 years, SNAQ: 11)

#### Eating to survive

Attitudes to appetite loss also differed. with some participants describing appetite only as a physical need to ‘fuel the engine’,

*“I eat to live; I don’t live to eat… I know I have to eat food to live… I know that I’ve got to, at least eat two meals a day because I feel strange, I feel light headed…but I’m eating food because I’ve got to… I suppose you could class it as being the same as putting petrol and oil in your car, you want to go from A to B so you put some petrol in, I want to do something so I eat” (*Male,79 years, SNAQ: 13,)

For these participants much less thought was given to food or to eating for pleasure.

*“If it’s fuel for the engine I don’t always enjoy that, I get something for dinner because I feel I really need to get something for dinner…I eat because I need to”* (Female, 76 years, SNAQ: 10)

#### Practical strategies

The differing perceptions and attitudes to appetite loss impacted on eating patterns and practical strategies used to cope with it. Some participants described very clearly the magnitude of the challenges faced.

*“It’s difficult…because* [husband] *might sit and he’ll put, not a big dinner out but…I think it’s big, and when I look at it, I could vomit, and I think how…am I going to get that down? And then he gets annoyed…because I’m not eating properly and so it goes on…”* (Female, 87 years, SNAQ: 8)

Others had practical strategies that they found helpful, although eating could be viewed as a ‘chore’ that had to fit into daily routines, often describing cooking as requiring ‘too much effort’ and preferring to ‘graze’ food or snack.

*“I wouldn’t consider I have proper times of meals; I graze…”* (Female, 82 years, SNAQ: 12)

Linked to changes in meal patterns, many participants opted for smaller portion sizes, describing larger portions as ‘off-putting’ or ‘nauseating’.

### Theme 3: living with poor appetite—the role and importance of the eating environment and social interactions

The eating environment and social context of mealtimes were described by the participants as important influences on the desire to eat. Social interactions appeared to be closely linked to appetite, both in impacting on appetite loss (Theme 1) and in supporting participants to cope with their poor appetite.

#### Eating environment

Eating with others was often seen as an opportunity to socialise which, in turn, could have a positive effect on appetite and encourage greater food consumption.

*“When I go out for a meal with people, before the Covid, it is a long time you’re sat at the table, you’re waiting for it to come, you’re talking, you are comparing what you’re having ‘oh that looks nice maybe I’ll have that next time…”* (Female, 61 years, SNAQ: 13)

#### Social context

A lack of social interaction, particularly for participants who cooked for themselves and ate alone, was therefore seen as a factor that could contribute to poorer appetite. This was highlighted both by participants who lived alone and for participants who ‘grazed’ over the day and no longer had structured mealtimes.

*“It depends on the mood and the effort…the effort part… think ‘oh I can’t be bothered to chop up the onions? can I be bothered to do this and do that? especially if you’re cooking for one person”* (Female, Age 61, SNAQ: 13)

Eating alone required effort and motivation whereas eating with others contributed to food enjoyment.

*“If somebody came in and put a meal in front of me, I would eat it…if I go out to dinner…if somebody else prepares the food or I’m in a restaurant my appetite’s absolutely fine…social activity I suppose, if I’m eating socially my appetite improves, I would never dream of having three courses if I’m at home but I can manage three courses in a restaurant or eating out with friends”* (Female, Age, 76, SNAQ: 10)

Importantly, many participants described the positive effects of food being prepared for them, being more likely to look forward to a meal and to have improved appetite.

## Discussion

The aim of our study was to develop a better understanding of appetite loss in older age. Using qualitative methodology, we conducted semi-structured interviews with a group of older adults with poor appetite, to explore their perspectives and experiences of appetite and appetite change, and to find out about the contexts of those experiences. To date, qualitative research in this area has focused on malnourished and hospitalised older adults—to our knowledge this is the first qualitative study of a group of older community-dwelling adults who have poor appetite, and it contributes novel data to current evidence on the lived experience of appetite loss.

We identified three themes from our interviews that provide key messages to take forward. The first of these messages is the need to recognise the wide range of influences on appetite and appetite loss in older age, and their complex interactions, as an essential consideration in the design and development of future strategies to support older adults. Participants linked factors from biological, psychological, and social domains to changes in appetite, at the same time, describing marked differences in the balance and role of specific causal factors in individual ‘journeys’ to poor appetite. Most commonly, participants identified more than one influence contributing to their appetite loss, also highlighting the overlap between domains and that many influences were often interrelated. This web of influences (Fig. 1) aligns with previous quantitative studies that have identified different types of factors linked to appetite loss, that include sociocultural, health-related, psychological and social factors [3, 21]. For example, emotional well-being and depression and social changes, including living and eating alone and retirement, have been linked to poor appetite [3, 8]. However, much of this evidence is cross-sectional and provides limited insights into individual experiences of appetite loss or the temporal sequence of events in older age. This has been a limitation of quantitative research to date; there is a need for different approaches in future studies, to enable descriptions of the ‘way’ appetite is lost [13].

In comparison, outside the setting of hospital care and treatment for malnutrition, there has been less qualitative research on appetite in older age. The experience of appetite loss therefore remains poorly described, although the findings of some qualitative studies on age-related changes in food intake and dietary habits resonate with those of the present study—also indicating a range of influences, that include biological, psychological and social factors [5, 6]. The most relevant evidence comes from a recent qualitative study of older outpatients attending a hospital clinic in the south of England. Although only four of the thirteen participants in this study had poor appetite, there are many consistencies with the present study, also describing the wide range of influences on appetite loss, that align with the biopsychosocial model for health and disease, recognising the wide range of biological, psychological, and social factors that influence health [22],which is also helpful in aiding understanding of the complex influences on appetite loss in older age and how they interact to result in distinct individual narratives of appetite loss [13].

The second key message is that there appear to be marked differences in perceptions of appetite loss and attitudes to poor appetite in older age, and that these differences impact on practical coping strategies. An important novel finding from our study is that for many participants, there was a reluctance to recognise their appetite loss and/or to accept that it was poor. This can be compared with the study by Cox et al. [13] in which variation in perceptions of appetite was also reported and linked to differences in adaptive behaviours. However, as the majority of the participants in that study had normal appetite, their experiences of appetite change are likely to have been more mixed. In contrast, all participants in the present study had poor appetite, as defined by the Simplified Nutritional Appetite Questionnaire [17]. Further research is needed to confirm whether the lack of awareness of appetite loss, or reluctance to accept that appetite is poor, is also prevalent in other settings, as this finding has obvious implications for preventive and treatment strategies. At present, appetite is not routinely assessed in clinical practice, with a recent report concluding that this has resulted in appetite loss being under-evaluated and under-treated [23]. Routine assessment should enable early identification of appetite change and therefore expected to be important for the development of effective preventive strategies in the future.

The third key message is that social interaction and the eating environment are important influences on appetite in older age. In particular, the role of social interaction was highlighted by participants—both in narratives of appetite loss that were attributed to changes in social circumstances, and in the management and coping strategies adopted when appetite was poor. This is consistent with the findings of previous quantitative and qualitative studies, and points to their inclusion in intervention strategies to support older adults, as modifiable factors that could be addressed [6, 24]. Efforts to support social interaction should also impact positively on linked psychological factors, such as wellbeing and mood, that were highlighted as key influences on appetite by the participants in our study. For example, a recent mediation analysis of cross-sectional data indicated a positive association between social activities and appetite in a group of older community-dwelling adults, but that this was indirect, and mediated by having fewer depressive symptoms [21]. Upstream interventions that promote social interaction and other community-based activities in older populations offer major potential in targeting the negative effects of social frailty and supporting and promoting health [25] and should be a priority for assessment in future research.

Importantly, while we identified three themes that were distinct, there was also some overlap between them which may have implications for strategies to support older adults. For example, the role of social interaction and positive eating environment was common to the narratives of appetite loss (Theme 1) and important for participants’ coping mechanisms (Theme 3). Additionally, psychological factors, such as low mood, appeared to impact both on appetite loss (Theme 1) and on attitudes and perceptions to it (Theme 2). Although further research is needed to explore and understand these interrelationships better, it highlights the potential of interventions targeting social and psychological factors that could be effective for practice, both for prevention and treatment of appetite loss.

### Strengths and limitations

A main strength of our study was the use of an on-line version of a widely used screening tool [17] which enabled us to focus on the narratives of older adults in the community, all of whom were living with poor appetite, to provide new data on the lived experience of appetite loss. All interviews were conducted by the lead author, an experienced health psychologist, and followed a semi-structured format. The researcher had no previous experience of poor appetite in older adults. The analysis was rigorous and went through many iterations, with the themes developed in consultation with the PPI group and with other authors before being organised in a thematic map. Discussing the themes with the PPI group enabled diverse perspectives to inform the development of the themes and the interpretation of the data.

However, while we feel confident that these themes are valid and meaningful, we recognise that the size of the sample and the mode of recruitment (on-line) may limit the wider generalisability of our findings and we did not conduct further member checking. A particular limitation of this study is that the participant interviews were conducted in 2021, following a phased ending to a series of ‘lockdowns’ in the UK that had been imposed as part of public health measures to minimise the impact of the COVID-19 pandemic. During these periods of lockdown, social interactions outside the home were not permitted at all or were restricted—with greater emphasis put on higher-risk groups, that included older adults. While the lockdowns may not have impacted on the biological influences on appetite that were described by the participants, they are likely to have been very important in their effects on psychological wellbeing, linked to the lack of social interaction, which was not possible during some of those periods [26].

Although freedom of movement had been restored by the time we conducted the interviews, continued anxiety around the pandemic was ongoing, particularly among older adults, and likely to have impacted on our findings. We are not able to assess the effects of the pandemic on the narratives of appetite loss in this study, and further research may be needed to revisit these themes in a post-pandemic setting.

## Conclusions

A complex range of influences and experiences of appetite loss in older age were described in this study, that require careful consideration in the design of effective prevention and treatment strategies. A lack of awareness or reluctance to accept appetite loss among some of our participants also has important implications. Future research should consider person-centred approaches, that take account of individual narratives of appetite loss, in the design of effective strategies to support older adults.

### Supplementary Information

**Additional file 1: Supplementary Table 1.** Topics and questions - guide for semi-structured interview.

## Data Availability

The dataset generated and analysed during this study includes potentially identifiable/confidential patient information and is not publicly available. Further information is available from the corresponding author on reasonable request.

## Abbreviations

SNAQ: Simplified Nutritional Appetite Questionnaire
PPI: Patient and Public Involvement
UK: United Kingdom
