# Supplementary material for: Exploring the experience of appetite loss in older age: insights from a qualitative study
Source: BMC Geriatr. 2024 Jan 31;24:117. doi: 10.1186/s12877-024-04732-9 (PMC10829396; doi:10.1186/s12877-024-04732-9)
Supplement: Supplementary file 1 — Additional file 1: Supplementary Table 1. Topics and questions - guide for semi-structured interview. [file 12877_2024_4732_MOESM1_ESM.docx]

**Supplementary Table 1. Topics and questions - guide for semi-structured interview.**

| **1. Appetite**  When I say appetite, what does that mean to you?  Could you tell me about your appetite?  How much thought do you give to your appetite?  Can you describe what you are thinking or feeling leading up to a meal?  What would you say makes you feel hungry?  What would you say makes your appetite worse (or reduces your hunger)?   - Is there anything that makes your appetite better?   How would you describe your motivation towards eating?   - Is there a difference in how motivated you feel to prepare food versus eating food? |
| --- |
| **2. Appetite change**  How do you feel about your current level of appetite?  Can you describe how your appetite has changed over time?   - When did you notice a change in your appetite? - What do you think has caused this?   How do you feel about having poor appetite?   - How do you manage this?   Can you describe a typical of day of when you eat/mealtimes? (fixed/flexible)   - How has this changed over time?   Can you describe how pleasant or unpleasant the eating experience is to you?  Can you describe your mood and general well-being and if this affects your appetite? |
| **Social Context**  Can you tell me a little bit about your living arrangements?   - Who do you usually eat with? - How often do you eat with others? - Does this impact on your appetite? |
